# Exosome Secretion — More Than Simple Waste Disposal? Implications for Physiology, Diagnostics and Therapeutics

**DOI:** 10.5772/62975

**Published:** 2016-04-01

**Authors:** Sivappriyan Nagarajah

**Affiliations:** 1 University of Oxford, Oxford, UK

**Keywords:** Exosomes, Physiology, Disease, Diagnostics, Diagnosis, Therapeutics, Treatment

## Abstract

Less than 100 nm in size and spherical in form - exosomes – vesicles expelled and taken up by cells, have ignited a new-found fascination. One which is derived from the sheer variety of exosomal content, ranging from microRNAs to transcription factors, capable of affecting a multitude of processes and pathways simultaneously within a target cell. Initially dismissed in 1983 as a waste disposal mechanism, today they form an entire field of research, being documented thus far in invertebrates, mammals, pathogens and potentially some plants. Many studies have suggested these spherical enigmas may possess a function, being implicated in processes ranging from animal behaviour to viral infection. This review will evaluate the evidence for the role of exosomes in physiology and pathophysiology, as well as their potential for application in the diagnosis and treatment of disease.

## 1. Introduction

Within our bodies, there exist minute space shuttles, making contact with unknown lands and, in the process, reshaping them. This is the world of exosomes - bubble - like spheres that bud off from cells containing a concoction of lipids, proteins and genetic material as their cargo. Less than 100 nm in size, these vesicles possess the potential upon landing to not only alter a target cell's metabolism but ultimately function [1]. Initially reported in red blood cells [2] and once dismissed as mere ejection pods removing cellular waste, today they stand at the forefront of research into intercellular, interorganismal and interspecies communication [1, 3, 4]. Documented thus far in mammals [5–7], invertebrates [8–9], pathogens [10] and potentially some plants [11], exosomes offer a way for the world around us and ourselves to interact, both in health and disease [12]. Indeed, the processes in which they are implicated reflect this, ranging from animal behaviour [13] to viral infection [14]. Their presence in a variety of bodily fluids, from cerebrospinal fluid to urine, make them readily accessible, be it for study or as diagnostic tools [15]. As decoding of exosomal signalling advances, what does this hold for our understanding of disease and our attempts to alleviate it?

Exosomes form one part of a larger universe - that of extracellular microvesicles (MVs), which are broadly divided into exosomes, ectosomes and apoptotic vesicles. Ectosomes are vesicles that directly budd off from the plasma membrane without involving the endocytic pathway, whilst apoptotic vesicles are remnants of cells that have undergone programmed cell death [12]. This review will primarily focus on exosomes, as many studies have indicated that they may possess a biological function [10, 13, 14, 16–21], as well as being useful in diagnosis [15] and treating disease [22].

Importantly, many exosome studies rely on vesicle size and biomarkers to fractionate and identify exosomes – a crude technique at best, which may mean that investigators are often studying a much more complex mixture of extracellular vesicles. However, the findings from these studies, the questions they raise and their implications, if true, make them highly significant.

**Figure 1.**
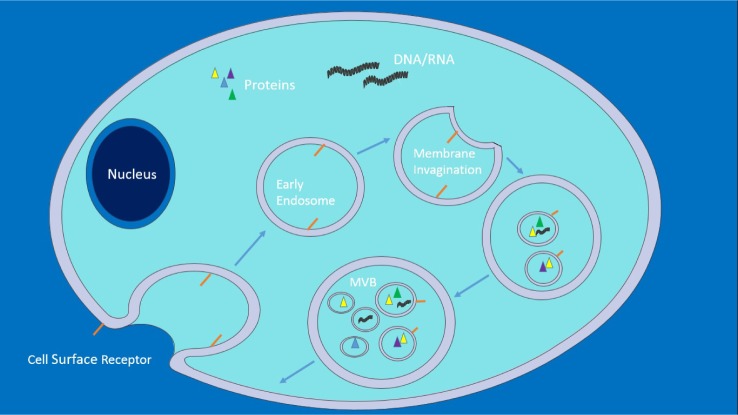
This depicts early endosome invagination from the plasma membrane of a cell. As the endosome matures to become a late endosome, endosomal membranes invaginate forming ILVs, giving rise to an MVB. Proteins from the Golgi complex can join the endosome prior to ILV formation as well as after. MVBs can then fuse with a lysosome, which enables proteasomal degradation, or with the plasma membrane releasing ILVs – now termed exosomes – into extracellular space. Adapted from[32],this figure was produced using Servier Medical Art, available from www.servier.com/Powerpoint-image-bank.

One must first look at exosomal biogenesis [12, 23–28] (Fig. 1) in order to recognize the normal state of affairs and how this provides avenues for exploitation, both therapeutically and by pathogens. Within a cell, as part of the endocytic pathway, a region of the plasma membrane invaginates, forming a vesicle that enters the cytoplasmic space, carrying with it cell surface receptors and extracellular fluid – an early endosome. As endosomes mature, they become more acidified and act as a sorting site, recycling certain proteins whilst targeting others for degradation. In these late endosomes, known as multivesicular bodies (MVBs), invaginations of the endosomal membrane also occur, forming numerous intraluminal vesicles (ILVs) that contain cytoplasmic content, such as short interfering RNA (siRNA). MVBs can then fuse with a lysosome, where proteins are degraded, or with the cell's plasma membrane, releasing the ILVs – now known as exosomes – into the extracellular space. The power of exosomes as signalling agents arise from the sheer variety of the contents they carry – capable of affecting a multitude of processes and pathways simultaneously within a target cell: from microRNAs, which prevent translation of mRNAs [29], to proteins, which can act as transcription factors [30] or even emit oncogenic phenotypes [31].

**Figure 2.**
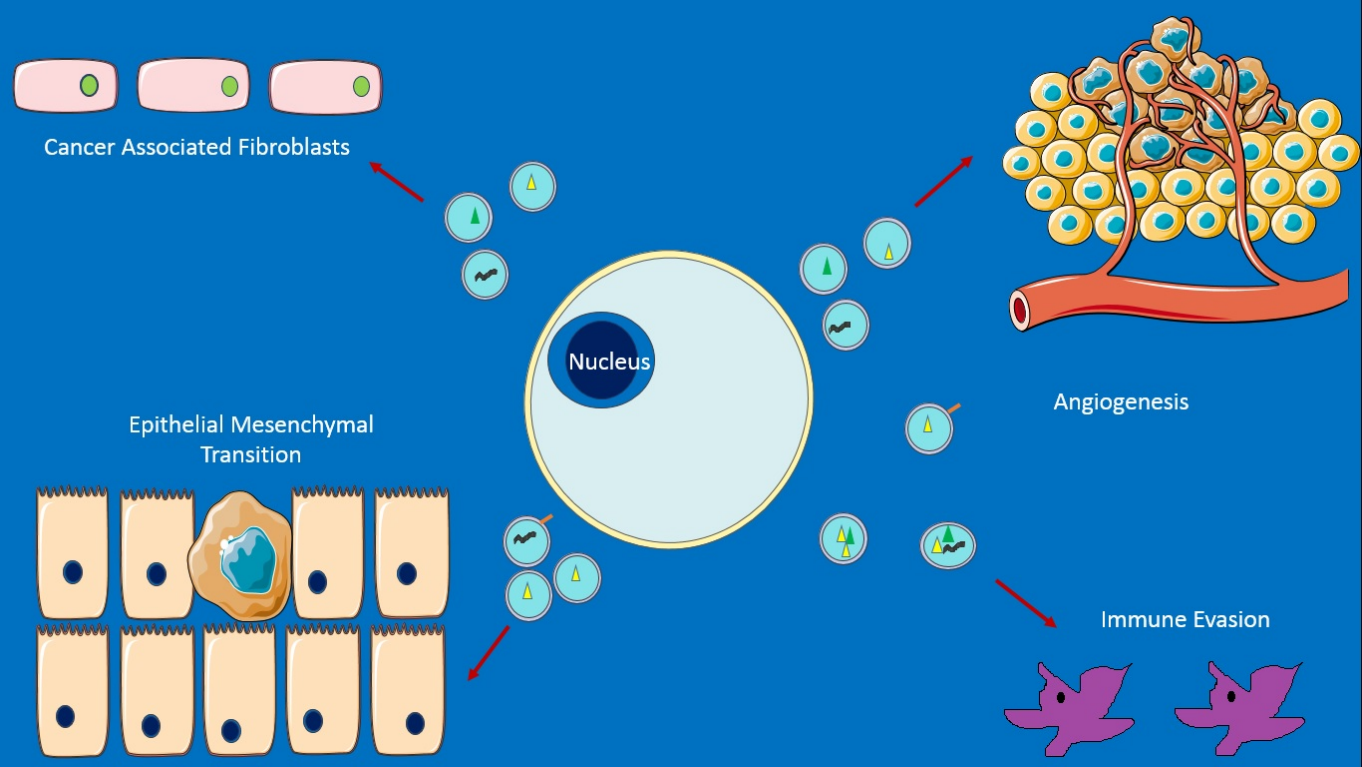
Some emerging roles of exosomes in cancer. Top left: promotion of nearby cells to act like fibroblasts secreting and sheathing the cancer in a fibrous layer that acts as a barrier, e.g., to drugs. Top right: promotion of new blood vessel growth and metastasis. Bottom left: aiding epithelial-mesenchymal transition (EMT) – with cells becoming capable of expressing a variety of proteins, as well as leaving the original site. Bottom right: enabling survival from immune response. Exosomal miRNA may be an important contributor to all of the above. Adapted from [33]. This figure was produced using Servier Medical Art, available from www.servier.com/Powerpoint-image-bank.

## 2. Exosomes in Physiology and Pathophysiology

### 2.1 Cancer

Exosomes appear to act as vehicles of transmission when hijacked. Perhaps the most well-known case is cancer, in which exosomes are thought to contribute to the creation of a microenviromental niche that promotes cancer cell survival (Fig. 2), as well as reprogramming distant tissue for invasion [33]. Take, for example, exosomes from Epstein Barr Virus (EBV) transformed lymphoblastoid B cells, containing miRNA: when exposed to dendritic cells (DCs), they lead to dose-dependent suppression of the immunoregulatory gene CXCL11/ITAC - known to be a target of EBV in promoting lymphomas. The peripheral blood of patients with EBV reveals EBV miRNA to be present in non–B cell populations, unlike EBV-DNA, which further suggests that miRNA transfer occurs *in vivo* [34].

Moreover, we are beginning to unveil how exosomes may mediate metastasis. Injection of the fluorescently labelled pancreatic cancer cell line - PAN02- derived exosomes, has confirmed their ability to increase the metastatic load in the liver of mice [35]. Other pancreatic cell-line-derived exosomes, including human BxPC-3 exosomes, also display a preference for the liver. Subsequent experiments have demonstrated that exosomal uptake by Kupffer cells leads to TGF-β synthesis and release, activating hepatostellate cells that go onto express fibronectin. This, then, attracts bone marrow-derived macrophages and granulocytes, which are now determined to be a prerequisite for metastasis, opening an avenue for future work.

However, in both cases it is important to note that arbitrary exosomal concentrations were used, sometimes *in vitro* and further research is needed to establish what truly occurs *in vivo.*

Nonetheless, when considered with studies illustrating exosomal explusion of cancer drugs [36–39], exosome-induced agiogenesis [40–43] and promotion of fibroblast differentiation and fibroblast-like cell formation, which sheaths the caner in a fibrous layer acting as a barrier (e.g., to drugs) [44–46] – exosomes add to the Darwinian paradigm of cancer cells as an evolving agent, with those capable of manipulating the cell machinery gaining a clear advantage in self- propagation.

### 2.2 HIV

HIV dissemination also involves rerouting of cellular behaviour. In which case, could exosomes play a similar role here? Conventionally, infected immature DCs are thought to travel to the lymph node, where they mature to interact with CD4+T cells, transferring the infection and resulting in CD4+ T-cell depletion with accompanying catastrophic immunodeficiency [48]. Recent work, which pulsed HIV-1 with DCs, showed not only increased exosomal release from infected DCs, but also that these exosomes promoted apoptosis when exposed to CD4+ T-cells [14]. How infection can be maintained, when given the short half-lives of CD4+ T-cells (less than two days), remains a puzzle. HIV is known to be able to enter DCs via endocytosis (transinfection), rather than direct fusion with the plasma membrane. The Trojan exosome hypothesis, therefore, proposes that HIV enters DCs in this alternative fashion, becoming part of the endosome and being later released into the extracellular space with exosomes. This means that CD4+ T-cells, which are rapidly transcribing and translating HIV, and are soon likely to die, can transfer HIV to DCs, which frequently interact with naïve CD4+ T-cells, thereby maintaining the infection. Confocal microscopy and various other imaging techniques have shown, via visualization, that the above is certainly possible *in vitro*, while *in vivo* effects remain to be established.

### 2.3 Physiology

Just as exosomes can become agents of dissemination, they may otherwise act as agents of control within the body when unaffected. Presentation of MHC-peptide complexes or antigens by DC-derived exosomes is capable of eliciting antigen-specific immune responses from other DCs [1]. The effect of exosomes appears to be dependent on the cell type of origin and the physiological state of the said cell type. Whilst exosomes from DCs can be immunostimulatory, cancer cell exosomes contain both cancer cell antigens, that could potentially be used to activate the immune system, and immunosuppressive molecules *in vivo*. Establishing which of the aforementioned effects is more dominant remains to be determined. When DCs are exposed to immunosuppressive chemicals or altered in order to transcribe immunosuppressive cytokines, this change in their physiology appears to promote tolerance by the exosomes secreted instead.

Whilst roles in immune regulation emerge, exosomes are also known to be expelled by neurons [49], microglia [20] and possibly adipose tissue [50] – could any regulatory role extend beyond immunity to territories, such as neuroendocrine physiology? In unravelling exosomal physiology and pathophysiology, we currently lack an established model to study exosomes *in vivo*. This is further complicated by our current techniques for selectively enriching and studying exosomes *in vitro* being too reliant on size and biomarkers - features no longer accepted as effective in completely removing other microvesicles from the sample [12]. Are any of the effects seen *in vitro* truly due to exosomes, or even reflective of physiological concentrations and their effect? One way of overcoming this hurdle would be to knockout genes known to be involved in exosomal regulation in a simple invertebrate model, such as *C. elegans*. These knockouts can be both systemic and tissue-specific in order to determine any functional consequence. One group of candidates for this would be the ESCRT proteins, which are known to regulate MVB and ILV formation [12, 51]. However, as they are also involved in degradation of ubiquitylated proteins, when a MVB fuses with a lysosome, protein turnover could be affected. Experimental models, therefore, would need to take this into account and distinguish any effects due to changes in exosomal biogenesis from the latter.

**Table 1.** Examples of body fluid exosomal markers (specifically, some of the proteins and RNAs) associated with pathology. Modified from [15, 52]

Source:	Disease	Proteins	miRNA	Ref
				
CSF	Alzheimer's Disease	AT270+ Phosphorylated Tau		[53]
Plasma	Chronic Hepatitis C	CD81		[54]
	Melanoma	CD63, caveolin-1, TYRP2, VLA-4, HSP70, HSP90		[55,56]
	Breast Cancer	Glypican -1	miR-141, miR195	[57,58]
	Pancreatic Cancer	Glypican -1		[57]
	Prostate Cancer		miR-16, miR-34b	[59]
	Premature Birth	Fas ligand, HLA-DR, CD3-zeta, IAK3		[60]
Urine	Bladder Cancer	EGF receptor pathway proteins		[61]
	Liver Injury	D26, CD81, Slc3A1, CD10		[62]
	Renal Fibrosis		miR-29c	[63]
				
Ascites	Ovarian Cancer	L1CAM, CD24, ADAM10, EMMPRIN,		[64]

Despite the concerns raised above, perhaps the strongest evidence for exosomal functionality *in vivo* would be work done on *Drosophila melanogaster* [13]. Secondary cells of male accessory glands, equivalent to the prostate, were shown to release GFP-tagged exosomes in to the seminal fluid, which not only “fuse with sperm *in vivo*” but also interact with the epithelium of the female genital tract, specifically reducing remating in females and, thus, providing an evolutionary advantage to the male *Drosophila*. When exosomal production is blocked in secondary cells, via removing proteins required for exosomal biogenesis, either by RNA interference or dominant negative RAB11 expression, 50–60% of males were unable to prevent remating in females, compared to 18% in the control group. It is remarkable to consider that something as minute as an exosome may bring about a change in something as biologically complex as behaviour. Furthermore, the observation of exosomal fusion with sperm is concordant with *in vitro* studies on human sperm, where prostate-derived exosomes (prostasomes) contribute to sperm motility [16].

## 3. Exosomes as Diagnostic Tools

From a diagnostic point of view, it does not matter whether exosomes have a function or not – only that a significant difference exists, either in composition or presence in both sickness and health, with these differences then being specific and attributable to different ailments. Indeed, this appears to be the case for many pathological conditions, with both exosomal content and presence varying [12] (Table 1).

One interesting study concerns exosomes fractioned from the blood of pregnant women with no history of any previous pregnancies at 28–30 weeks of gestation [60]. Significant differences existed between those going on to deliver at term and those delivering prematurely: specifically, lower levels of FAS L, HLA-DR and reduced inhibition of JAK3 and CD3 zeta in premature births. Could an exosomal screening programme be the future? Given the small sample size, more work is needed before any clinical translation can occur.

In cancer, however, the use of exosomes in diagnostics appears more imminent. Exosome Diagnostics have developed a “urine-based liquid biopsy” which looked at three specific exosomal RNAs prior to prostate biopsy in 195 men. It was found that high grade cancer could be predicted based on a score derived from RNA levels alone, with a sensitivity of 95.2% and a negative predictive value of 97.5% [65]. Exosome Diagnostics points to the use of a first catch urine sample and a single score that can prevent unnecessary biopsies in low grade tumours as key advantages. Additional research looking at economics and outcomes is underway, as well as expansion into “blood plasma-based liquid biopsy” [66]. Further excitement in this field arrived with a recent attempt to detect cancer exosome specific biomarkers. Primary work on cancer cells and other non-cancer lineages using mass spectrometry suggested 48 proteins [57]. Of these, glycoprotein glypican – 1 (GPC1), which, although found circulating at low levels in healthy patients sera, was higher in patients with breast cancer (n = 32, 75%) and pancreatic ductal adenocarcinoma (PDAC, n = 190, 100%). Analysis of a small group of patients revealed that GPC1 levels distinguished precursor lesions (n=5) from benign pancreatic disease (pancreatitis & cystic adenomas, n = 18) - something that could not be done by the current tumour marker for pancreatic cancer, CA-19-9. ROC curve comparison of CA-19-9 and GPC-1 showed the latter to be superior at all stages of PDAC with 100% sensitivity and specificity, as well as positive and negative predictive value. Whilst the prospect of an early exosomal marker for pancreatic cancer is joyous, the nature of the small cohorts in this work means that further confirmation is required.

Exosomes offer three key advantages as a diagnostic tool: they are less invasive compared to a biopsy, they can be used where a biopsy is not possible (e.g., brain tumour) and may become cost-effective and time-efficient [67, 68].

Although the most common techniques of exosomal fractioning, which are ultracentrifugation and density gradient separation can require over five hours [69], work is already underway to overcome this. One promising avenue is microfluidic-based exosomal detection, which is essentially a “lab on a chip” [68]. The prototype consists of various chambers, inlets and a microchannel within a polydimethylsiloxane chip, enabling integration of several processes. This includes exosomal isolation and enrichment, as well as the detection of biomarkers from minute volumes of plasma (30 μl). Initially, a plasma sample is mixed with magnetic beads coated in antibodies to desired surface markers, and injected via an inlet. A magnetic field is then applied, trapping the exosomes bound to the beads, with subsequent washing in a PBS solution. Following this separation and enrichment, incubation with a lysis buffer, which is inserted via another inlet, releases exosomal content. Next, the lysate moves through a winding channel, with magnetic beads coated in antibodies flowing from the two side channels, targeting inter-exosomal content of interest. Bead-inter-exosomal content is then trapped in a chamber via a magnetic field, where chemifluorescence detection can be carried out, meaning levels of specific inter-exosomal content can be measured. This technique not only takes one and a half hours, but was also successful in identifying the insulin-like growth factor 1R (IGF-1R) – a potential biomarker for non-small cell lung cancer (NSCLC), in plasma of patients with NSCLC. It is also possible to embed exosomes extracted in this manner and view them under transmission electron microscopy, thereby enabling higher levels of characterization. With greater mapping of disease-specific exosomal markers, the use of microfluidic technology, such as this routinely, may become a reality.

## 4. Exosomes as Therapeutic Tools

One of the features that makes exosomes useful in diagnostics also means they can be useful therapeutically. Essentially, exosomal surface markers can be used to target specific cell types. Be it for drug delivery or gene therapy, exosomes engineered and secreted by a chosen cell type can be packaged with desired components [22] and administered by a simple injection [70]. Another appealing feature is the use of patients' own cells to generate exosomes in order to enable biocompatibility [22]. Although therapy is the most ambitious and fledgling aspect in exosomal research, its untapped potential is worth considering.

Take the brain for instance - one of the most difficult places to deliver any kind of therapy within the human body. This is due to the blood-brain barrier, restricting what may and may not enter, in an attempt to protect the neural cavity [71]. However, exosomes are known to be released by neurons within the brain parenchyma [34], as well as transport pathological agents, such as alpha–synuclein between neurons in Parkinson's [17]. Recently, rat choroid plexus-derived exosomes were shown to distribute folate - vital for DNA synthesis via cerebrospinal fluid [19]. Work on C57BL/6 mice removed their bone marrow, selecting immature DCs known to produce copious amounts of exosomes that are non-T-cell stimulatory [70]. These cells were then transfected with plasmids expressing targeting peptides; in the case of the brain, these were rabies virus glycoprotein (RVG). This led to formation of exosomes with desired surface markers. Next, electroporation was adapted and applied at the nanoscale in order to enable exosomal uptake of exogenous SiRNA. RVG9R exosomes were also created with the addition of nine D-arginines to the RVG, which interacted electrostatically with loaded SiRNA. SiRNA to BACE1 (a key gene in Alzheimer's) containing RVG exosomes were injected into C57BL/6 mice. Controls included untreated mice and mice injected with RVG9R–BACE1. Three days later, cortical tissue analysis revealed significant protein knockdown (45%, P < 0.05, versus 62%, P < 0.01) when RVG9R-BACE1 and RVG-BACE1 were used, respectively. β-amyloid 1-42 levels, an important constituent of amyloid plaques, fell by 55% (P <0.01) in mice treated with RVG exosomes, whilst serum markers of an inflammatory response (IFN - α, TNF – α, IL - 6, IP-10) were not increased. The ability to cross the blood-brain barrier is perhaps a pharmacological holy grail; like the holy grail, however, whether one should obtain it remains a question. Can we treat in the vicinity of such a delicate and vital organ? Although the same question could have been asked at the advent of bypass surgery, which is now almost routine.

Moreover, in situations such as immune-mediated loss of beta cells of the pancreas (Type I diabetes) [72] or scarring of cardiac tissue post-myocardial infarction (MI) [73], a promising regenerative strategy has been stem cell implantation [74]. However, in some cases at least, it may be the exosomes released by stem cells that deserve credit. For example, a recent study showed that cardiosphere-derived stem cells (CDCs), when infused via coronary circulation in post-MI patients, led to decreased scar formation and increased viable mass at six months [75]. More interestingly, when CDCs were inhibited from releasing exosomes in a murine model of MI, they were unable to reproduce the above effects. Furthermore, CDC-derived exosomes alone could produce the regenerative effects displayed by CDCs [18]. This offers a potential cell-free approach to regenerative medicine, if applicable to other stem cells.

Another avenue is the use of exosomes as vaccines [76–78]. Here, the ability of mature DCs to create immunostimulatory exosomes could be exploited, via presenting DCs with pathologic antigens before injection of generated exosomes. Largely thus far explored in cancer, its application could be broader if successful. Work using DCs, which are pulsed with tumour antigens as cancer vaccines, appears to be more potent at generating anti-tumour immunity than other techniques (e.g., viral vaccines). It remains to be seen whether exosomes can match, if not better, this. Equally, in the treatment of autoimmune disease, the creation of immunosuppressive exosomes by altered DCs may be useful [1].

## 5. Conclusion

What were once dismissed as waste disposal agents have now become an entire field of study [1]. With an evergrowing list of potential applications and investment opportunities, our knowledge regarding extracellular communication is likely to expand in the next decade. Success will rely crucially on the development of an *in vivo* model for determining exosomal function. Whilst the current work on therapeutics and diagnostics is promising, much remains to be seen as to how it will play out clinically. Will it be safe? Will we be able to transform concept into reality? Despite the gathering interest, numerous unknowns remain. However, this should not be a hindrance to progress – if anything these questions ought to dictate further research and exploration into a tantalizing field.
